# Symptoms of mental illness among university student-athletes during the second wave of the COVID-19 pandemic lockdown in Canada

**DOI:** 10.3389/fspor.2022.1017376

**Published:** 2022-10-19

**Authors:** Véronique Boudreault, Sophie Labossière, Véronique Gauthier, Sophie Brassard, Sophie Couture, Frédérick Dionne, Catherine Laurier, Natalie Durand-Bush

**Affiliations:** ^1^Faculté des Sciences de l'Activité Physique, Université de Sherbrooke, Sherbrooke, QC, Canada; ^2^Faculté d'Education, Université de Sherbrooke, Sherbrooke, QC, Canada; ^3^Département des Sciences Humaines, Université du Québec à Trois-Rivières, Trois-Rivières, QC, Canada; ^4^School of Human Kinetics, University of Ottawa, Ottawa, ON, Canada

**Keywords:** collegiate student-athletes, dual role, mental disorders, motivation, stress

## Abstract

The COVID-19 pandemic lockdown disrupted the university sports season and had negative consequences on the academic and personal life of university student-athletes, resulting in several psychological challenges. The goal of this study is to document the symptoms of mental illness among university student-athletes during the second wave of the COVID-19 pandemic lockdown in Canada. It aims to (a) assess the prevalence of mental illness symptoms (anxiety, depression, disordered eating, and dangerous drinking) among university student-athletes and (b) identify which sociodemographic and sports characteristics, pandemic impacts, and levels of perceived stress most influence these symptoms. A total of 424 university student-athletes completed an online survey, which included questions on mental illness and the impact of the pandemic lockdown. The results revealed a notable prevalence of the symptoms of mental illness; depressive symptoms are reported by 37.9% of the participants, anxiety symptoms by 24.9%, dangerous drinking symptoms by 10.1%, and disordered eating by 8.6%. In addition, being female [OR = 0.56, 95% CI (0.33, 0.95)] or a member of a visible minority group [OR = 2.63, 95% CI (1.02, 6.78)] are significantly associated with the presence of depressive symptoms. Low academic motivation has a significant negative influence on the presence of depressive [OR = 3.37, 95% CI (1.82, 6.25)] and anxiety symptoms [OR = 2.75, 95% CI (1.35, 5.62)]. However, the presence of perceived stress was strongly associated with depressive [OR = 7.07, 95% CI (3.26, 15.35)], anxiety [OR = 6.51, 95% CI (3.30, 12.84)], and dangerous drinking symptoms [OR = 5.74, 95% CI (2.51, 13.14)]. This study advocates for specific mental illness prevention and treatment resources tailored to the unique needs of university student-athletes. Accordingly, partnerships and practical interventions to support university student-athletes' mental health are presented.

## Introduction

Data available on the mental health of higher education students show signs of important psychological impairment during the pandemic (1–3). A worldwide meta-analysis of 89 studies assessing depressive and anxiety symptoms among higher education students during the pandemic (from January 2020 to January 2021) showed high prevalence of depressive (34%) and anxiety (32%) symptoms (4). The elevated level of psychological distress in higher education students could be attributable to the impacts of the pandemic on the academic motivation and social life of these students (5, 6). The prevalence of mental illness varies from country to country. It is affected by many factors including the time of data collection, the country's preparedness to respond, its economic vulnerabilities, the diagnostic criteria used for measuring mental illness as well as physical epidemics, and multiple other psychosocial factors related to cross-cultural differences (4, 7).

For university student-athletes playing a dual role (that of university student and elite athlete), the disruption to both training and competition activities during the various waves of the pandemic might have produced additional stressors. Physical activity, social connection, and team cohesion are fundamental aspects of academic sports participation and are known to be beneficial for the mental health of these students (8). Concerns that could have negatively impacted the mental health of university student-athletes include sadness over the loss of competitive seasons, reduced social support networks, lack of engagement in training, fear of contracting COVID-19, isolation, academic issues, and financial concerns (9–11). For many student-athletes, sports engagement and athletic identity are strongly associated with academic motivation and success (12).

Data collected in studies conducted before the pandemic show prevalence rates of anxiety and depression at around 15–30%, with the highest rates among female athletes (13, 14). In a cross-sectional study of 437 student-athletes of the National Collegiate Athletic Association Division (NCAA) before and after the postponement of the fall 2020 sports season due to the COVID-19 pandemic lockdown, 20% of the sample endorsed specific symptoms such as feelings of agitation, difficulty winding down and relaxing, overreacting to situations, and lack of initiative (15). Among student-athletes competing in Division II and III of the NCAA and National Association of Intercollegiate Athletics (NAIA) during the COVID-19 pandemic lockdown, Grauspenberger et al. (16) found a prevalence rate of 27.4% of depressive symptoms (measured with the eight-item depression scale from the Patient-Reported Outcomes Measurement Information System) and a high level of COVID-19-specific worrying. At the beginning of their final year, Japanese university student-athletes reported a high level of hopelessness resulting from a decrease in motivation due to sports cessation during the COVID-19 pandemic lockdown (17). The scant available data show a sharp difference in the prevalence rates of anxiety and depressive symptoms (ranging from 12.1 to 27.4%), but generally suggest a negative impact on student-athletes' wellbeing.

To date, descriptive studies have mostly assessed anxiety and depression symptoms while disordered eating and dangerous alcohol use among university student-athletes during the lockdown have been overlooked. Alcohol abuse is a frequent mental illness reported by collegiate and university student-athletes (18), and disordered eating is more prevalent among athletes than in the general population (19). These problems often coexist with depression and anxiety (20). In addition, the consumption of psychoactive substances, relapses, and the development of substance use disorders in the general population increased during the lockdown (21, 22). Guillot et al. (23) found that 16.7% of female and 15.2% of male university student-athletes were classified as hazardous drinkers in April and May 2020, and males reported significantly higher alcohol consumption than females. Also, higher rates of unhealthy eating habits and preoccupations with eating and body image were reported in both the general population (24, 25) and among athletes (26). To better understand the mental illness symptoms experienced by student-athletes, studies should consider symptoms such as dangerous drinking and disordered eating symptoms in addition to symptoms of anxiety and depression.

Chronic stress, life events, and their physiological impacts may predispose certain people to anxiety, depression, dangerous drinking, and disordered eating (27, 28). It is conceivable that the pandemic adds to the stressors already experienced by university student-athletes, resulting in environmental challenges that may exceed their ability to cope,. The developmental transition to adulthood (e.g., acquisition of financial and emotional autonomy, departure from the family home), concerns about their university studies (e.g., higher workload, the need to acquire advanced writing and critical thinking skills), and their participation in elite sports (e.g., lack of time to develop a social network outside of the sports field, sports retirement) are among the stressors they experience (29–31). All these stressors (developmental, academic, athletic, and pandemic) are relative. In contrast to absolute stressors, such as earthquakes or shootings, relative stressors may or may not be perceived as stressful (32).

Given that exposure to these potential stressors did not automatically result in mental illness, it is also important to assess perceived stress. Although the relationship between perceived stress and the development of mental illness symptoms is under-researched among university student-athletes, it has been more widely studied among their non-athlete peers. Several pre-pandemic studies found that university students with higher levels of perceived stress had more depressive and anxiety symptoms (33, 34). Among university students, stress caused by the lockdown was associated with a greater likelihood to binge eat and restrict food intake in the week before the study and intentions to do so in the 15 days following it (35). The influence of perceived stress on mental illness symptoms needs to be assessed in university student-athletes to better understand the impact of stress on their mental condition.

In an editorial paper published in the *British Journal of Sports Medicine (BJSM)*, Grubic et al. (8) discuss the lack of attention paid to the impact of the pandemic on student-athletes' mental health, pointing out that their risk of developing mental illness is higher than that of non-athlete students. However, despite the unique reality and needs of student-athletes, compared with professional/elite athletes and non-athlete students, studies on the mental condition of student-athletes during the COVID-19 pandemic lockdown are still scarce. To respond appropriately and provide mental health support and tailored approaches to care, it is important to gather information about mental illness issues experienced by university student-athletes during the lockdown along with the consequences on their lives.

Until now, most studies examining the mental health of student-athletes during the pandemic have been conducted in the United States [e.g., (15, 16, 23, 36)], the situation of Canadian university student-athletes has therefore been understudied. It is worth mentioning that there are major differences in the Canadian university sports context compared to the NCAA context in terms of available funding, quality of sports infrastructure, as well as the level of sport played (37). While in the United States it is possible to receive athletic scholarships that cover all costs (housing, food, tuition, textbook costs, weekly allowance), student-athletes at Canadian universities can only rely on scholarships that fully or partially cover their tuition (37, 38). Moreover, in the province of Quebec (Canada), 49.5% of university athletes are gainfully employed (39), as they cannot count on the same financial support as in the United States. Also, Canadian university student-athletes generally play in front of much smaller crowds than their American counterparts, their competitions are rarely televised, and media attention is less common than in the United States (40). Besides these differences, the disparities between the education and health care systems in Canada and the United States are significant enough to preclude generalizing the results of the United States studies to the Canadian university student-athlete population (14). Finally, Canadian university sport is not based on the same levels of athletic competition as the NCAA (Division I, II, and III). Student-athletes who join a varsity team all have the level of competition required to play in the varsity league: they have been recruited by the coaches or have passed the athletic tests. In addition to varsity teams that compete between universities at the provincial and national level, there are also intramural teams, in which the level of competition is truly recreational. For the purposes of this article, the focus is solely on student-athletes who are part of a Canadian varsity team.

Therefore, the objective of this study is to document the symptoms of mental illness among university student-athletes during the second wave of the COVID-19 pandemic lockdown in Canada (in the fall of 2020). It aims to (a) assess the prevalence of the symptoms of mental illness (anxiety, depression, disordered eating, and dangerous drinking) among university student-athletes and (b) identify the sociodemographic characteristics, pandemic impacts, and levels of perceived stress that have the greatest influence on the symptoms of mental illness. The hypothesis is that university student-athletes for whom the COVID-19 pandemic lockdown had the greatest negative impacts on their sports and academic life, and who report a high level of perceived stress, will experience more symptoms of anxiety, depression, dangerous drinking, and disordered eating. By compiling a portrait of the mental condition of university student-athletes in the province of Quebec (Canada), this study aims to motivate university decision-makers to put in place specialized resources to support these athletes' mental health.

## Materials and methods

### Participants

University student-athletes in the province of Quebec, Canada were asked to complete an online questionnaire for this research. Participants had to be a member of a Quebec university sports team for a sport affiliated with the Quebec Student Sports Network (Réseau du sport étudiant du Québec) for the 2020–2021 season. The final sample included 424 Quebec university student-athletes from 12 Quebec universities. Of this number, 319 participants fully completed the questionnaire, which resulted in variations in the number of participants per analysis.

### Measures

Sociodemographic and Sports Characteristics questions about gender, sex, sexual orientation, ethnicity, visible minority status, and age were presented at the beginning of the questionnaire. Next, participants were asked about their sports and academic background, their main sport, the number of academic years completed, and their home university. They were asked if they had ever been diagnosed with a mental disorder and if they were currently using prescribed medications to treat mental illness issues. Finally, they were asked about their current participation in sports (e.g., “I no longer train in my university's facilities”; “I cannot practice my sport due to public health measures related to COVID-19”).

*Generalized Anxiety Disorder* [AD-7; (41)] is a seven-item self-report measure that assesses symptoms of generalized anxiety (41). The frequency of symptoms during the past 14 days is scored on a four-point scale from “0: never” to “3: almost daily.” The overall score (the sum of the seven items) can be anywhere between 0 and 21. The higher the score, the more severe the anxiety symptoms. A high level of anxiety symptoms is indicated by an overall score of 10 or greater (41). This test has good internal consistency (α = 0.92) and test-retest reliability (*r* = 0.83), and is recognized as a valid, reliable, and consistent measure of generalized anxiety disorder symptoms (41). This questionnaire was also validated in a national student-athlete NCAA population and has good psychometric qualities (42). In the context of this study, the internal consistency of the French and English versions of this questionnaire is also very good (α = 0.89 and α = 0.88).

*Patient Health Questionnaire-9* [PHQ-9; (43)] is a nine-item self-report measure about the symptoms of depression. The frequency of symptoms during the last 14 days is scored on a four-point Likert scale ranging from “0: never” to “3: almost daily.” The overall score (the sum of the nine items) can be between 0 and 27, the higher the score, the more severe the depressive symptoms. A important level is indicated by an overall score of 10 or higher. This English questionnaire has good psychometric qualities, with internal consistency and test-retest reliability of, respectively, α = 0.89 and *r* = 0.84 (43), and the French version is comparable (44). This questionnaire was also validated in a division II student-athlete NCAA population and has good psychometric qualities (45). In the context of this study, the internal consistency of the French and English versions of this questionnaire is also satisfactory (α = 0.82 and α = 0.86).

*The Alcohol Use Disorders Identification Test-Concise* [AUDIT-C; (46)] is a three-item, short-form self-report measure that screens for alcohol use disorders in the last 12 months. Each item is measured on a five-point scale ranging from 0 to 4. The overall score (the sum of the three items) ranges from 0 to 12, with overall scores of five and above for women and seven and above for men indicating the presence of dangerous drinking. The AUDIT-C has good test-retest reliability (*r* = 0.88) and internal consistency (α = 0.76) with good psychometric qualities (47). This questionnaire has not yet been tested with an athletic population (48), but validation with university students has supported good psychometric qualities (49). In this study, the internal consistency of the French and English versions of the AUDIT-C is fair (α = 0.61 and α = 0.66).

*The Eating Attitudes Test*–*26* [EAT−26; (50)] is a 26-item self-report measure that screens for eating disorders (bulimia and anorexia). Items 1–25 are scored on a six-point scale ranging from “0: never/rarely/sometimes” to “3: always.” The 26th item is scored on a reversed scale. The overall score (the sum of the 26 items) varies between 0 and 76. A score of 20 or higher is associated with a greater risk of developing an eating disorder. The English and French versions of this tool have good psychometric qualities; good internal consistency, discriminant validity, and factorial structure (50, 51). This questionnaire was also validated with a female student-athlete population and has good psychometric qualities (52). In this study, the internal consistency of the French and English versions of the EAT−26 is very good (α = 0.89 and α = 0.92).

*The Perceived Stress Scale* [PSS −10; (53)] is a 10-item self-report measure that assesses perceived stress during the past month. This is defined as perceiving a situation to be threatening or demanding and, at the same time, taxing in terms of resources. Perceived stress is scored on a five-point scale ranging from: “0: never” to “4: very often.” The overall score (the sum of the 10 items) can be between 0 and 40. A score of 13 or under indicates a low level of perceived stress. A score between 14 and 26 indicates a moderate level, and a score of 27 or above indicates a high level. The French and English versions of PSS −10 have good psychometric qualities, namely good internal consistency (α = 0.81 and α = 0.73) and good factor validity (53, 54). This questionnaire was also validated with a student-athlete population and has good psychometric qualities (55). In the context of this study, the internal consistency of the overall PSS −10 score of the French and English versions is very good (α = 0.86 and α = 0.86).

*The Pandemic Impacts Questionnaire COVID-19* included questions designed by the research team to address the personal reality of each university student-athlete during the pandemic. This questionnaire included sections about: (a) their participation in sports, (b) the impact of the COVID-19 pandemic lockdown on their different spheres of life, and (c) the classification of each sphere according to how much it contributed to the overall feeling of stress. In the first section, questions were asked about sports participation (e.g., “I no longer train in my university's facilities”–yes/no; “I cannot practice my sport due to public health measures related to COVID-19”–yes/no) and the sports retirement plans (e.g., “I want to end my sports career earlier OR later OR no change”) and the pandemic alert level in their region (e.g., red, orange, yellow, or green). In the second section, questions were asked to assess the impact of the COVID-19 pandemic lockdown on the 13 spheres of life: training motivation, competition motivation, academic motivation, mental performance skills practice, financial situation, self-esteem, physical condition, relationships with coaches, relationships with teammates, relationships with loved ones (family, friends, and lovers), stress management, psychological wellbeing, and professional career plans. The participants had to indicate the extent each sphere was affected on a five-point Likert scale. The Likert scores were converted into dichotomous scores (presence or absence of a negative impact of the pandemic on each of the spheres of life). The goal of the questions in the third section was to classify each of these spheres of life according to its contribution to the overall feeling of stress (e.g., studies, sport, work, financial problems, family, love life, time constraints/lack of time, and health).

### Procedure

Data for this study were collected from October 14 to December 8, 2020, using an online questionnaire available on LimeSurvey (version 3.25.10+210128). The research team sent emails to the athletic director and coordinators at each university and provincial sports association (Quebec Student Sports Network and Quebec Foundation for Athletic Excellence). The recipients, in turn, forwarded the email invitation to the student-athletes in their organization, who could access the online questionnaire by clicking on a secure web link. The questionnaire (lasting ~30 min) was available in English and French. Before they could access it, participants had to read the consent form, which explained the terms of the project (benefits, risks, confidentiality, objectives), and then voluntarily and freely consent to take part in the study. Two separate ethics committees approved this project: the Research Ethics Committee–Education and Social Sciences of the Université de Sherbrooke (Ref. No.: 2020-2722) and the Human Research Ethics Committee of the Université du Québec à Trois-Rivières (Ref. No.: CER-20-271-10.01). Each participant had a 1 in 25 chance of winning a $25 gift card.

### Statistical analyses

To answer first objective, descriptive analyses were performed on anxiety, depression, disordered eating, and dangerous drinking symptoms. Four multiple binary logistic regression analyses were run to address the second objective and to determine which characteristics have the greatest influence on the symptoms of mental illness. The characteristics included sociodemographic and sports information (gender, age, visible minority, type of sport, international student status, number of years as a university student-athlete, and pandemic zone), pandemic impacts and levels of perceived stress for each of the four mental illness (i.e., the presence of symptoms of depression, anxiety, dangerous drinking, and disordered eating). To reduce the number of variables in the regressions and to avoid type I errors, a bivariate-level screening of sociodemographic and sports variables significantly correlated with the presence of the symptoms of mental illness was performed (56). Only three of these variables (gender, visible minority, and sports type) were significantly (*p* ≤ 0.05) associated with the presence of depressive symptoms and were consequently included in the binary logistic regressions with depressive symptoms. No sociodemographic and sports variables were included in the other three logistic regressions, due to lack of a significant association with the other dependent variables (presence of major symptoms of anxiety, dangerous drinking, and disordered eating). The presence of high-perceived stress, pandemic impacts (the presence of negative pandemic disruptions on training motivation, competition motivation, and academic motivation), and sociodemographic and sports variables (if applicable) were included as independent variables in all four regressions.

Because these analyses are exploratory and were intended to identify the independent variables with the greatest influence on the presence of the symptoms of the four mental illness (dependent variables), the stepwise method (forward selection–conditional) was chosen (56). This method retains the variables that contribute the most to the model and excludes those that contribute the least. This helps explain the greatest amount of variance in the dependent variable using the fewest predictors. It is thus the most parsimonious models that have been retained (56). Finally, bivariate correlation analyses were performed to verify the assumptions of the multiple binary logistic regressions and to ensure that there was an absence of multicollinearity between the independent variables. The Hosmer and Lemeshow test was used to ensure that the models fit the data well (absence of significant deviation between the postulated relationships and the observed scores). All analyses were performed using IBM^®^ SPSS^®^ software version 25.0.0.2 (57). The 95% confidence level was selected (*p* ≤ 0.05) to reject the null hypothesis.

## Results

Supplementary Table 1 presents the sociodemographic and sports characteristics of the sample. The average age was 21.83 (varying from 18 to 43, *SD* = 2.60). Of the respondents, 62.7% identified as women, 37.0% as men, and 0.2% as other. Most were Caucasian (88.7%) and French-speaking (85.3%). A minority (12.7%) had been given a diagnosis of a mental disorder. Respondents spent an average of 14.06 hours per week in their varsity sport (*SD* = 7.50, *Mdn* = 12.00), and 66.2% were in paid employment during the school year, working an average of 14.15 h per week (*SD* = 9.21, *Mdn* = 12). At the time of their participation in this study, 71.1% lived in a red zone (maximum pandemic alert), 27.0% in an orange zone (moderate pandemic alert), 1.6% in a yellow zone (early pandemic warning), and 0.3% in a green zone (pandemic vigilance). Each color corresponds to a public health emergency with specific public health measures. Most participants lived in a maximum alert zone (red), where the main measures were the ban on gatherings, the closure of restaurants and fitness facilities, the obligation to wear masks, and the ban on playing sports and engaging in recreational activities. For participants in an orange zone, the main measures were a restriction on the maximum number of people allowed in private gatherings (six) and public activities (25) along with physical distancing measures. Because non-essential travel between regions was restricted, competitions were canceled.

Regarding the impact of the COVID-19 pandemic lockdown on their different spheres of life, 70.9% of participants reported that the COVID-19 pandemic lockdown had a negative effect on their academic motivation. In terms of sports, 55.6 and 70.6% reported a negative impact of the pandemic on their motivation to compete and their motivation to train, respectively. Many respondents reported a slight to very negative impact on their psychological wellbeing (72.8%); stress management (59.9%); physical condition (56.3%); mental performance skills practice (52.6%); relationships with teammates (44.7%); self-esteem (38.7%); relationships with loved ones, including family, friends and lovers (36.1%); professional career plans (36.1%); financial situation (31.6%); and relationships with coaches (18.3%).

### Snapshot of the symptoms of mental illness among university student-athletes

Notable prevalence rates of the symptoms of mental illness were observed among university student-athletes. As shown in Table 1, symptoms of depression were the most common, followed by symptoms of anxiety. Comparative analyses based on various attributes of university student-athletes (sex at birth, visible minority, and type of sport) were also conducted (see Supplementary material).

**Table 1:** Prevalence of mental illness symptoms among university student-athletes.

**Prevalence of mental illness symptoms**	**Percentage (frequency)**
Presence of symptoms of:
Depression	37.9 (132)^a^
Anxiety	24.9 (85)^b^
Dangerous drinking	10.1 (31)^c^
Disordered eating	8.6 (27)^d^

The prevalence rates presented are based on the cut-off scores of the questionnaires used. ^a^*n* = 348, ^b^*n* = 342, ^c^*n* = 306, ^d^*n* = 314.

### Contribution of sociodemographic and sports characteristics to symptoms of mental illness

To address the second objective of identifying the sociodemographic and sports characteristics, pandemic impacts, and levels of perceived stress with the greatest influence on the symptoms of mental illness, four stepwise multiple binary logistic regressions were conducted. To explain the presence of depressive symptoms in the first analysis, several variables were removed from the model because they did not contribute significantly (*p* > 0.05): the type of sport (individual or team), the six changes to sports participation, and the presence of pandemic-related negative impacts on training and competition motivation. The remaining variables: sex at birth, being a visible minority, having high perceived stress, and experiencing a negative impact on academic motivation (in contrast to an impact on training and competition motivation) statistically explained 23.4% of the variance in the presence of depressive symptoms (Table 2). Regarding the sociodemographic variables, being a visible minority increased the risk of having depressive symptoms by 2.63 times [95% CI (1.02, 6.78)], while being male decreased it by 1.78 times [95% CI (0.33, 0.95)]. Among the pandemic variables, the presence of high-perceived stress was associated with a 7.07 times higher likelihood [95% CI (3.26, 15.35)] of experiencing depressive symptoms, while having experienced a negative impact on academic motivation, due to the pandemic, was associated with only a 3.37 times higher likelihood [95% CI (1.82, 6.25)]. Thus, high perceived stress is the variable that best explains the presence of depressive symptoms.

**Table 2:** Multiple binary logistic regression of the presence of depressive symptoms.

	**Odds ratios (standard errors)**	**Confidence intervals (95%)**
Sex at birth	0.56 (0.271)*	0.330: 0.954
Visible minority	2.63 (0.482)*	1.023: 6.775
Presence of high perceived stress	7.073 (0.395)***	3.259: 15.352
Academic motivation	3.370 (0.315)***	1.817: 6.250
Constant	0.214 (0.300)	
Classification ratio	70.8	
*R* ^2^	0.234***	
*N*	322	

Entries correspond to standardized regression coefficients (odds ratios) with standard errors in parentheses. The presence of depressive symptoms was dichotomized based on the PHQ-9 threshold score (≥10–43). ***p ≤ 0.001, *p ≤ 0.05.

Next, to explain the presence of anxiety symptoms, the six changes to sports participation and the presence of negative impacts of the pandemic on training and competition motivation were removed from the model because these variables did not contribute significantly to the results (*p* > 0.05). Having high perceived stress and experiencing a pandemic-related negative impact on academic motivation statistically explained 18.7% of the variance in the presence of anxiety symptoms (Table 3). The presence of high perceived stress is associated with a 6.51 times higher likelihood [95% CI (3.30, 12.84)] of experiencing anxiety symptoms, while having experienced a pandemic-related negative impact on academic motivation increased this risk 2.75 times [95% CI (1.35, 5.62)]. Thus, in the model, the presence of high perceived stress is the variable that best explains the presence of anxiety symptoms.

**Table 3:** Multiple binary logistic regression of the presence of anxiety symptoms.

	**Odds ratios (standard errors)**	**Confidence intervals (95%)**
Presence of high perceived stress	6.507 (0.347)***	3.298: 12.841
Academic motivation	2.750 (0.365)**	1.345: 5.623
Constant	0.110 (0.336)***	
Classification ratio	79.0	
*R* ^2^	0.187***	
*N*	324	

Entries correspond to standardized regression coefficients (odds ratios) with standard errors in parentheses. The presence of anxiety symptoms was dichotomized based on the GAD-7 threshold score (≥10–41). ***p ≤ 0.001, **p ≤ 0.01.

To explain the presence of dangerous drinking behavior, the six changes in sports participation and the presence of negative impacts of the pandemic on training motivation, competition motivation, and academic motivation were removed from the model because these variables did not contribute significantly (*p* > 0.05) to the results. Having high perceived stress statistically explained 10.4% of the variance in the presence of dangerous drinking (Table 4). The presence of high perceived stress increased the risk of dangerous drinking 5.74 times [95% CI (2.51, 13.14)]. Thus, the presence of high perceived stress is the variable that best explains the presence of dangerous drinking.

**Table 4:** Multiple binary logistic regression of the presence of dangerous drinking symptoms.

	**Odds ratios (standard errors)**	**Confidence intervals (95%)**
Presence of high perceived stress	5.738 (0.423)***	2.505: 13.142
Constant	0.075 (0.244)***	
Classification ratio	90.0	
*R* ^2^	0.104***	
*N*	299	

Entries correspond to standardized regression coefficients (odds ratios) with standard errors in parentheses. The presence of dangerous drinking symptoms was dichotomized based on the AUDIT-C threshold scores [five and above for women and seven and above for men – (46)]. ***p ≤ 0.001.

Finally, none of the variables included in the model (the six changes to sports participation, the presence of high-perceived stress, and the presence of pandemic-related negative impacts on training, competition, and academic motivation) contributed significantly (*p* > 0.05) to explaining the variance in the presence of disordered eating.

## Discussion

This study is the first to focus on the mental condition of university student-athletes during the second wave of the COVID-19 pandemic lockdown in the province of Quebec, Canada. This exploratory research identified variables associated with the symptoms of mental illness, based on cross-sectional data. The first objective of this study was to assess the prevalence of the symptoms of mental illness (anxiety, depression, disordered eating, and dangerous drinking) among university student-athletes. The results point to noteworthy prevalence rates. Coherently with literature, depressive symptoms are the most prevalent, followed by anxiety symptoms. The prevalence found in similar sample [27.4%; (26, 36)] lower than that observed in the present study (37.9%), can be explained by the use of different tools for measuring depressive symptoms. Bullard' (36) and Bullard et al.'s (26) research on student-athletes competing in the Division III New Jersey Athletic Conference noted sadness, loneliness, irritability, crying, fatigue, and sleep disturbances. Regarding anxiety symptoms, in the United States during the fall of 2020, Schary and Lundqvist (58) found that 36.4% of their division I NCAA student-athletes had a clinical level of anxiety (using the Hospital Anxiety and Depression Scale). Their results reveal a higher prevalence of anxiety symptoms than those of this study (24.9%). The discrepancy may be explained by the difference in instruments used and the public health measures in the two countries (59). Further, the prevalence of dangerous drinking within the present study (10.1% of the sample) is lower than that identified in the study by Guillot et al. (23), which observed a prevalence of 16.7% among females and 15.2% among males. This discrepancy could be explained by sample differences, but there is a lack of information on the population studied (e.g., mean age and percentage of participants practicing an individual sport vs. a team sport) to confirm this. The context also differed between the two studies because of differences between the public health measures in place in the United States and Canada, and the divergent sport and university contexts (14, 37, 40, 59).

Predominant rates of depressive symptoms found among student-athletes in this study may be due to discouragement caused by sports being canceled for the second time, and isolation related to virtual classes and confinement. As shown in other studies involving student-athletes from the United States and Canada, being away from teammates, not having the necessary resources to train and to study, and social distancing were reported by participants as the most challenging impacts (16, 60). Symptoms of depression and anxiety are generally lower in student-athletes than non-athlete students, which emphasizes the role that sport can play in preserving mental health (61). Therefore, depriving student-athletes of this central area of focus in their lives may put them at risk of distress.

The second objective was to identify the sociodemographic characteristics, pandemic impacts, and level of perceived stress with the greatest influence on the prevalence of symptoms of mental illness. Results show that several factors influence the presence of depressive symptoms. Being female or a member of a visible minority group significantly increased the risk. This is not surprising. Depressive symptoms are known to be more common among women than men (62); however, the difference is usually not significant among university students and student-athletes (4, 63). Gender differences were significant in this study, but the difference in the risk of having important level of depressive symptoms was quite small. Being male decreased the risk by only 1.79 times [OR = 0.56, 95% CI (0.33, 0.95)], an observation that might not have been significant with smaller samples (and therefore less statistical power). Previous studies have recognized the difficulties faced by student-athletes from minority groups, such as a sense of rejection by teammates or classmates, feeling exploited for their athletic abilities, and feeling judged unfairly by their university administrations (64, 65). Additionally, student-athletes from visible minorities reported having access to fewer opportunities than their white teammates (e.g., for playing star positions, such as quarterback and taking on key roles in leadership), and to less academic guidance (e.g., lacking recommendations for course selection or advice for academic development purposes) (65). They also felt more targeted for “random” drug testing than did their white teammates (64, 66). Such additional stressors increase the demands on student-athletes of visible minorities and may predispose them more to mental illness symptoms (67).

The negative effects of the pandemic on academic motivation have a significant influence on the presence of depressive and anxiety symptoms in university student-athletes. This finding is consistent with previous research that demonstrated a positive correlation between sports involvement and student-athletes' satisfaction with their overall college experience, their motivation to complete their academic program and to continue their studies (68). The public health measures imposed during the pandemic, such as virtual learning (69), may have contributed to the development of anxiety and depressive symptoms. Virtual university courses may have created several issues including difficulties concentrating (e.g., distractions at home), deficient course delivery (monotony of virtual courses: pre-recorded lectures with minimal interaction, limitations of practice exercises, reductions in teamwork, and canceled or postponed internships), and loss of contact with student peers (70–72). Such impacts on academic life, due to the pandemic, may have affected the academic motivation of university student-athletes, and, in turn, led to anxiety and depressive symptoms.

Perceived stress is the variable that contributes the most to the presence of depressive and anxiety symptoms and dangerous drinking. It can be hypothesized that perceived stress is one main reaction to the pandemic repercussions. In this study, disruptions in sports participation may have explained some of the variance in the presence of depressive symptoms. However, in the presence of perceived stress, this contribution disappeared. Thus, it is not solely the assessed pandemic impacts that explain mental illness symptoms; we must consider perceived stress. High perceived stress could therefore be the link between disruption in sports participation and the presence of depressive symptoms. According to Bullard (36), student-athletes reported that sports acted as a stress outlet for them and that this outlet was no longer available during the pandemic. Sports may act as a protective factor during times of increased anxiety, such as the COVID-19 pandemic lockdown (73, 74). Therefore, the negative impact perceived on sports participation may have removed this protective factor for student-athletes, who then experienced increased stress and tried to find another coping mechanism. The presence of dangerous drinking, noted in 10.1% of the participants, may imply that some participants used drinking as a means to cope with the increase in stressors and perceived stress (75).

None of the variables in the analysis were statistically related to the presence of disordered eating. However, the lack of statistics due to the small number of participants presenting eating disorders symptoms (*n* = 27) may have resulted in Type II errors (76). Furthermore, disordered eating may be better explained by individual (internal) variables, such as psychological characteristics vs. environment variables (external), such as common stressors experienced by university student-athletes. For example, over-control (perfectionism and asceticism) and media pressure to be thin (e.g., “I've felt pressure from TV or magazines to have a perfect body”) are two elements known to statistically predispose Quebec university student-athletes to disordered eating (39). During the pandemic, time spent on social media increased among young adults and may explain an increase in disordered eating (77). Subsequent research could compare the prevalence of disordered eating before and after the pandemic to see if it increased in student-athletes.

The scientific literature recognizes that social support is an important protective factor against the development of depression for university student-athletes (34, 78). Graupensperger et al. (16) found that college student-athletes who received more social support and maintained a stronger connection with teammates had better mental health during the pandemic. University student-athletes reported that when they returned to practicing sport, meetings with their teammates alleviated their anxious and depressive feelings (79). Public health measures restricting social contact probably contributed to the development of depressive symptoms in university student-athletes. Because the student-athletes have busy schedules (balancing studies, university sports, and sometimes a part-time job), they derive most of their social support in the context of their sports (31). The measures affecting the athletes' participation in sports in the presence of coaches and teammates may have deprived the athletes of the support they needed to cope with the disruptions caused by the pandemic, such as virtual teaching, the disruption of several sectors of economic activity in which students are generally involved (e.g., restaurants, tourism, and sales service), concerns related to their health and that of their loved ones, etc. (70–72). Further research could investigate the role of social support in the prevalence of the symptoms of mental illness among student-athletes.

### Contributions of the research to applied practice and recommendations for interventions

In the literature, most mental health care programs for university student-athletes have been developed and tested among student-athletes in universities in the United States [e.g., (80–84)]. However, athletic and academic cultures vary considerably between Canada and the United States (14), as do the education and health systems. This may affect the implementation or effectiveness of such programs in Canadian settings. Although inspired by American university culture, university sports in Quebec (Canada) have not achieved comparable fame or media attention and generate less revenue (40). A good example of this difference is the much higher funding allocated to university sports in the United States than in Canada (37). Another difference between Canada and the United States is that specialized mental illness care services for university student-athletes in Quebec are very limited. Given these differences, an expected outcome of this study was to help mobilize university stakeholders to put in place and adapt resources to support the mental health of student-athletes in Quebec. As such, a research-informed intervention initiative, psychosocial treatment services, and recommendations for interventions based on the results are presented.

#### The research-informed intervention initiative

Led by the research team, a research-informed intervention initiative in line with the present results was implemented in university settings. This intervention had two objectives: (1) to inform stakeholders, coaches, and student-athletes about the needs and issues experienced by university student-athletes in the province of Quebec, and (2) to put in place support resources that adequately meet the needs of student-athletes in a volunteer university setting. Related to the first objective, an infographic popularizing the salient results related to the reality of university student-athletes (prevalence of mental illness symptoms, perceived stress levels, and impacts of the COVID-19 pandemic on their lives) was produced and made available on social media in addition to being shared by the Research Chair in Safety and Integrity in Sport. This document effectively mobilized the university sports community in the province. The director of the provincial Student Sports Network was met with to discuss the picture captured by the study.

Regarding the second objective of this initiative, an ongoing multi-level intervention was implemented at the home university of one of the authors, aiming to promote student-athletes' mental health. To this end, the athletic directors of this university were met with and quickly mobilized to address the situation. The prevalence of mental illness among the participants that attend this university was established, allowing the researchers to precisely determine the needs in this sport environment. Further, in collaboration with the athletic directors, steps have been taken with an organization in the region that specializes in mental health intervention in academic contexts. Prevention and treatment interventions were carried out through this collaboration. First, from a systemic and empowerment perspective, meetings were set up involving the university's athletic management, two coaches, and two student-athletes in order to discuss what is already in place in the environment to promote mental health and to reflect on ways to further improve the promotion of mental health. Several actions have been identified to either continue or to implement such as monthly or quarterly meetings where coaches and athletes discuss sport goals and personal difficulties; the education of coaches on subjects aimed at improving athletes' wellbeing (e.g., mindfulness, time management, managing stress and anxiety); and the meeting at the start of the sports season to raise mental illness awareness and orientation regarding the help resources available at the university. In addition, for preventive purposes, a mental performance consultation service is now available to sports teams at this university, offered by a psychoeducator who specializes in sports (the second author of the study). This psychoeducator is currently taking steps to join the Canadian Sport Psychology Association. Indeed, several interventions, putting into practice concepts and techniques (e.g., stress and activation management) related to mental performance training in sport, have been found to prevent or effectively treat various mental illness symptoms and promote mental health in university student-athletes (81, 83, 85–91).

#### Psychosocial treatment services

Finally, a psychosocial treatment service aimed at supporting the adaptation and wellbeing of student-athletes experiencing difficulties has been set up in this university. This ongoing service is offered by the psychoeducator mentioned above. In addition, steps are being taken to establish an agreement between the sports department of this university and an association in the region to facilitate referral to specialized services (e.g., sports psychologists and mental performance consultants) free of charge for the student-athletes. From the beginning of its implementation, feedback from stakeholders, athletes, and coaches has been highly positive. To our knowledge, this research-informed applied intervention initiative is unique in the province of Quebec, and warrants further impact evaluation. A program evaluation research project intended to empirically assess the impacts and efficacy of the interventions implemented has been planned.

#### Recommendations for interventions

Because perceived stress has been identified in the present study as the variable that has the greatest power to explain the presence of anxious, depressive, and dangerous drinking symptoms, future interventions should aim to help student-athletes develop skills to better cope with stress. To date, there are several programs for the prevention and treatment of mental illness evaluated with the population of university student-athletes, such as the Female and Male Body Project (80, 82), Athlete Mindfulness (83), and The Optimum Performance Program in Sports—TOPPS (81). When tested, all of these programs have significantly reduced symptoms of mental illness in college student-athletes (80–83, 86, 90, 92–94). However, few of these programs specifically target perceived stress and stressors *per se*. In order to improve the prevention and treatment programs available, it could be interesting to add content aimed at reducing stress. For example, a recent program, the Scarlet and Grit Resilience Training Program (84), was implemented and tested to equip university student-athletes by developing skills to adapt to the stressors they encounter. Such a program, for which a session was held once a year for 4 years, seems to be promising, but perhaps its intensity should be adjusted (e.g., by holding sessions at shorter intervals or giving reminders and assigning homework to consolidate learning). Future evaluative studies should be carried out to assess the program's effectiveness in reducing and preventing the symptoms of mental illness. Expressive writing seems to be another promising practice for helping university student-athletes improve the perceptions of the various stressors they experience (95–97).

### Limitations

Although this study has provided a picture of the mental illness symptoms of university student-athletes, in addition to allowing the implementation of an intervention initiative to promote mental health and the development of recommendations for intervention in university student-athletes, it has limitations that are worth noting. Because of the cross-sectional design used in the present study, it is impossible to say whether the population was predisposed to perceived stress, lower motivation to study, or symptoms of mental illness, or if the symptoms were a consequence of the pandemic lockdown. The causality hypotheses will have to be tested with data from subsequent measurements following this study on Quebec university student-athletes. Moreover, it is common for various mental disorders to occur simultaneously (98). To test this possibility in university student-athletes, the differences between various mental illness profiles could be explored through latent profile analyses. Further research is needed to explore this avenue. Another limitation of our study is the attrition of some participants in their completion of the questionnaire, whether it was due to their busy schedules or their mental illness symptoms. For this reason, in this study, prevalence rates may be lower than what would otherwise be seen. In addition, participants with important mental illness symptoms may not have participated in this study, resulting in selection bias. The self-reported nature of the online survey may also lead to other biases, including misclassification bias, as responses rely entirely on the judgment and interpretation of the participants. In future studies, the relationship between athletic and academic stressors, perceived stress, and the symptoms of mental illness need to be explored longitudinally to better understand the mechanism by which mental illness symptoms arise in the pandemic context. In addition, the role of social support in this mechanism should also be explored to guide further intervention and prevention avenues.

## Data availability statement

The data analyzed in this study is subject to the following licenses/restrictions: The data is not public, but if someone requests it for a legitimate reason, it will be possible to share it (while respecting the anonymity of the participants, as agreed with the research ethics boards). Requests to access these datasets should be directed to veronique.boudreault2@usherbrooke.ca.

## Ethics statement

The studies involving human participants were reviewed and approved by two separate Ethics Committees: the Research Ethics Committee–Education and Social Sciences of the Université de Sherbrooke (Ref. No.: 2020-2722) and the Human Research Ethics Committee of the Université du Québec à Trois-Rivières (Ref. No.: CER-20-271-10.01). The patients/participants provided their written informed consent to participate in this study.

## Author contributions

VB contributed to the writing (introduction, methods, results, and discussion) and collected data (lead researcher). SL contributed to the writing (introduction, methods, results, and discussion), performed the statistical analyses, and collected data. VG contributed to the writing (methods) and collected data. SB contributed to the writing (discussion). SC, FD, and CL critically reviewed the study proposal and contributed to the data collection. ND-B contributed to the data collection. All authors contributed to the article and approved the submitted version.

## Conflict of interest

The authors declare that the research was conducted in the absence of any commercial or financial relationships that could be construed as a potential conflict of interest.

## Publisher's note

All claims expressed in this article are solely those of the authors and do not necessarily represent those of their affiliated organizations, or those of the publisher, the editors and the reviewers. Any product that may be evaluated in this article, or claim that may be made by its manufacturer, is not guaranteed or endorsed by the publisher.
