# Supplementary material for: Symptoms of mental illness among university student-athletes during the second wave of the COVID-19 pandemic lockdown in Canada
Source: Front Sports Act Living. 2022 Oct 19;4:1017376. doi: 10.3389/fspor.2022.1017376 (PMC9627313; doi:10.3389/fspor.2022.1017376)
Supplement: Supplementary file 1 [file Table_1.docx]

Supplementary Material

| **Table 1** Sociodemographic and Sports Characteristics of the Sample (*N* = 424) | | | | | |
| --- | --- | --- | --- | --- | --- |
| **Sociodemographic Variables** | | **Percentage (Frequency)** | **Sports**  **Characteristics**  **Variables** | | **Percentage (Frequency)** |
| Ethnicity  White  Black  Asian  Indigenous  Other | | 88.7 (375)  3.5 (15)  2.4 (10)  0.9 (4)  4.5 (19) | Main University Sport  Soccer  Volleyball  Ultimate  Rugby  Soccer  Cheerleading  Cross-country running  Swimming  Track and field  Badminton  Basketball  Ice Hockey  Other sports ^a^ | | 14.6 (62)  12.7 (54)  9.7 (41)  9.4 (40)  8.5 (36)  6.8 (29)  6.1 (26)  5.9 (25)  5.4 (23)  5.2 (22)  4.0 (17)  3.5 (15)  8.0 (34) |
| Presence of a diagnosed mental disorder ^a^ | | 12.7 (51) | Main sport type  Team  Individual | | 30.0 (127)  70.0 (297) |
| Visible minority  International student  First language  French  English  Other | | 7.1 (30)  4.5 (19)  85.3 (361)  10.4 (44)  4.3 (18) | Second university sport  No other sport  Athletics  Cross country  Soccer  Basketball  Volleyball  Other ^b^ | | 88.8 (373)  5.0 (21)  1.4 (6)  1.2 (5)  1.0 (4)  1.0 (4)  1.7 (7) |
| Medication for mental illnesses | | 7.7 (31) | Years completed in university sports  0  1  2  3  4  5 | | 30.7 (130)  25.5 (108)  21.0 (89)  12.8 (54)  9.2 (39)  0.7 (3) |
| ^a^ Mental disorder diagnosed during a lifetime  ^b^ Currently taking prescribed medication for a mental disorder | | | ^a^ Other Main Sports: tennis (n = 9), triathlon (n = 6), cross-country skiing (n = 5), golf (n = 4), cycling (n = 2), sailing (n = 2), ice canoeing (n = 2), boxing (n = 1), gymnastics (n = 1), synchronized swimming (n = 1), and alpine skiing (n = 1).  ^b^Other Secondary Sports: triathlon (n = 2), badminton (n = 1), baseball (n = 1), cheerleading (n = 1), rugby (n = 1), and alpine skiing (n = 1). | | |
| **Changes in Sports Participation During the Pandemic** | | | | **Percentage (Frequency)** | |
| 1 | My sports practice has changed since the pandemic began. | | | 97.0 (358)^1^ | |
| 2 | I no longer train in my university’s facilities. | | | 58.3 (215)^1^ | |
| 3 | I no longer train in my coach’s presence. | | | 64.3 (236)^2^ | |
| 4 | I no longer participate in competitions. | | | 93.2 (343)^3^ | |
| 5 | I no longer train with my teammates. | | | 50.1 (185)^1^ | |
| 6 | I cannot practice my sport because of public health measures related to the COVID-19 pandemic lockdown. | | | 49.6 (184)^4^ | |
| **Change in Sports Retirement Plans During the Pandemic** | | | | **Percentage (Frequency)** | |
| 1 | My retirement is earlier than expected. | | | 13.2 (49)^5^ | |
| 2 | My retirement is later than expected. | | | 17.0 (63)^5^ | |
| 3 | My retirement plans have not changed. | | | 69.7 (258)^5^ | |

# Note: Percentages (and frequencies) of participants who identified with these items. ^1^N = 369, ^2^N = 367, ^3^N = 368, ^4^N = 371, ^5^N = 370.

**Table 6. Comparative analyses based on various attributes of university student-athletes**

| **Presence of** |  | **Sex at birth** | | **Visible minority** | | **Type of sport** | |
| --- | --- | --- | --- | --- | --- | --- | --- |
|  |  | Female | Male | Yes | No | Individual sports | Team sports |
| **Depressive symptoms** | Percentage (frequency) | 42.6 (92) | 29.8 (39) | 59.1 (13) | 36.5 (119) | 30.4 (34) | 41.5 (98) |
|  | Pearson chi-square value (df) | 5.70 (1) | | 4.47 (1) | | 4.02 (1) | |
|  | *p* | .017 | | .035 | | .045 | |
|  | N | 347 | | 348 | | 348 | |
| **Anxiety symptoms** | Percentage (frequency) | 27.6 (59) | 19.7 (25) | 24.2 (78) | 35.0 (7) | 22.0 (24) | 26.2 (61) |
|  | Pearson chi-square value (df) | 2.67 (1) | | 1.17 (1) | | .69 (1) | |
|  | *p* | .102 | | .279 | | .407 | |
|  | N | 341 | | 342 | | 342 | |
| **Dangerous drinking symptoms** | Percentage (frequency) | 10.3 (20) | 9.9 (11) | 0.0 (0) | 10.1 (31) | 5.2 (5) | 12.4 (26) |
|  | Pearson chi-square value (df) | .01 (1) | | 2.03 (1) | | 3.72 (1) | |
|  | *p* | .923 | | .154 | | .054 | |
|  | N | 306 | | 306 | | 306 | |
| **Disordered eating** | Percentage (frequency) | 10.5 (21) | 5.3 (6) | 10.0 (2) | 8.5 (25) | 12.7 (13) | 6.6 (14) |
|  | Pearson chi-square value (df) | 2.53 (1) | | .05 (1) | | 3.31 (1) | |
|  | *p* | .111 | | .817 | | .069 | |
|  | N | 314 | | 314 | | 314 | |
| **High perceived stress** | Percentage (frequency) | 14.8 (31) | 12.1 (15) | 9.5 (2) | 14.4 (45) | 7.6 (8) | 17.0 (39) |
|  | Pearson chi-square value (df) | .49 (1) | | .38 (1) | | 5.27 (1) | |
|  | *p* | .484 | | .536 | | .022 | |
|  | N | 333 | | 334 | | 334 | |

**Note: Chi-square tests were performed; percentages are shown with frequency in parentheses. The cut-off scores of the questionnaires were used to assess the presence of each kind of symptoms. df = degree of freedom**
